# The scPagwas analysis of single-cell sequencing combined with Bayesian deconvolution with SMR Reveals Tumor Heterogeneity and Predicts Immunotherapy Response in Neuroblastoma

**DOI:** 10.7150/jca.138740

**Published:** 2026-09-28

**Authors:** Changlin Jia, Daowei Wang, Changfeng Jia, Pan Wang, Yun Liu, Qiang Zhao

**Affiliations:** 1 Department of Pediatric Oncology, Tianjin Medical University Cancer Institute & Hospital, National Clinical Research Center for Cancer, China.; 2 Tianjin's Clinical Research Center for Cancer, China.; 3 Key Laboratory of Cancer Immunology and Biotherapy, Tianjin, China.; 4 Department of Histology and Embryology, School of Basic Medical Sciences, Cheeloo College of Medicine, Shandong University, Jinan, 250012, China.; 5 Interdisciplinary Center, Shandong University, China.

**Keywords:** neuroblastoma, single-cell pathway-based genome-wide association study, bayesian deconvolution, immunotherapy response, summary-data-based Mendelian randomization

## Abstract

**Objective:**

High-risk neuroblastoma, the predominant driver of pediatric cancer deaths, is marked by marked intratumoral heterogeneity that fuels therapy resistance. To predict response to and targets for immunotherapy, we use single-cell and bulk RNA sequencing data to reveal tumor heterogeneity in neuroblastoma, and to identify prognostic genes for neuroblastoma.

**Methodology:**

Single-cell data were analyzed for trait-associated subpopulations and genes using single-cell Pathway-based genome-wide association study (scPagwas), and TARGET data were analyzed for differentially expressed genes. Bayesian deconvolution of trait-related subpopulations was performed to analyze the association between differences in convoluted cellular fractions, survival, and clinical traits. Further analysis of the association of convoluted cellular fractions with various immune-related gene sets with immunotherapy. Core gene validation survival, function, and correlation analysis of a convolutional cell-based screen for neuroblastoma. Summary-data-based Mendelian Randomization (SMR) was used to further validate the core genes.

**Results:**

Multiple cell subpopulations such as IL7R/CD4^+^ T cells were identified in neuroblastoma based on single-cell sequencing data. Among them, the FYB/CD4^+^ T cell subpopulation is strongly associated with multiple features in neuroblastoma. The convoluted cellular fractions obtained from this cell subpopulation also correlate with several features of neuroblastoma and can predict response to immunotherapy. Core genes from convoluted cell-based screens have been associated with a variety of traits including survival and cell function in neuroblastoma.

**Conclusions:**

Neuroblastomas have distinct cell subpopulations, and neuroblastomas deficient in the FYB/CD4^+^ T cell subpopulation have poorer survival and impaired immune cell infiltration and response to immunotherapy. Several genes, like *RGS1*, could play a central role in neuroblastoma development.

## Introduction

Neuroblastoma is a pediatric malignancy that arises from the neural crest-derived cells of the peripheral sympathetic nervous system. It is characterized by aberrant differentiation of sympathetic neurons, which is attributed to genetic and epigenetic alterations. Neuroblastoma is associated with mutations that impair the normal developmental processes of the sympathetic nervous system^1^. Neuroblastoma is the most common solid tumor in children, accounting for about 15% of deaths from childhood-associated cancers^2^. As medical research progresses, the overall survival rate for pediatric patients with low-risk neuroblastoma has exceeded 90%. However, for those with high-risk neuroblastoma, the survival rate remains below 50%^3^. Subsequent investigations reveal that neuroblastoma treatment outcomes are significantly correlated with tumor heterogeneity. This heterogeneity can be influenced by the age of the patient at the time of diagnosis, the presence of disease metastasis, and the intrinsic biological characteristics of the tumor^4^. Moreover, the poor prognosis of neuroblastoma treatment stems from acquired treatment resistance in a heterogeneous tumor microenvironment^5^. However, the heterogeneity of neuroblastomas remains elusive, highlighting the need for further investigation. At present, while Bulk RNA-Seq has been extensively applied in neuroblastoma research, it frequently falls short in capturing the nuanced heterogeneity of individual cellular compartments^2^. The emergence of single-cell RNA sequencing (scRNA-seq) has afforded an intricate, cell-by-cell perspective on the genetic and phenotypic terrain within tumors. This cutting-edge technology has been pivotal in elucidating the intricate heterogeneity inherent to cancer profiles^6^,^7^.

The emergence of single-cell sequencing technology has significantly augmented the synergy with genome-wide association studies (GWAS), thereby enhancing the elucidation of trait-associated genes. Therefore, Jianzhong Su team developed a new algorithm, single-cell Pathway-based genome-wide association study (scPagwas), a pathway-based multi-gene regression method, which combines GWAS data and scRNA-seq data to identify individual cells associated with traits, and can be more effective in identifying trait-related genes^8^. Furthermore, Charles G. Danko and colleagues developed an innovative algorithm that utilizes single-cell sequencing data as a priori information, enabling the precise prediction of cell type and gene expression in individual samples from extensive RNA sequencing data^9^.

In this study, based on scRNA-seq data and bulk RNA-seq data of neuroblastoma, we combined two advanced algorithms, scPagwas and Bayesian deconvolution, to individually assess the cell type and characteristic gene expression of each neuroblastoma patient for the first time, and revealed the heterogeneity among neuroblastomas. Furthermore, patients were predicted to respond to immunotherapy based on their cell type and screened for trait genes. The findings of our study contribute to a deeper understanding of the diverse genetic and phenotypic characteristics of neuroblastoma. Furthermore, our study offers novel insights into immunotherapy strategies that may enhance the efficacy and survival outcomes of neuroblastoma patients.

## Materials and Methods

### Data acquisition and processing

The scRNA-seq data for neuroblastoma were obtained from the Gene Expression Omnibus (GEO) database (GSE218450), which comprises a dataset described as single-cell data used to train the inverse convolution algorithm^10^. The data set comprising bulk RNA-seq and clinical information on neuroblastoma was sourced from the Cancer Genome Atlas (TCGA) database (https://portal.gdc.cancer.gov/). The GWAS data for neuroblastoma were obtained from the GWAS summary website (https://gwas.mrcieu.ac.uk/, ebi-a-GCST004885). The quantitative trait locus (QTL) data for the single-nucleotide polymorphism (SNP) analyses were obtained from the Summary-data-based Mendelian Randomization (SMR) website (https://yanglab.westlake.edu.cn/software/smr, Adrenal_Gland.zip). The reference data for the analyses were downloaded from the CNCR database (https://cncr.nl/research/magma/).

### scRNA-seq data analysis

The scRNA-seq data were initially processed using the R package Seurat 4.0, with the criterion of at least three cells expressing each gene and a minimum of 250 genes expressed per cell^11^. The percentage of mitochondria and rRNA is calculated by the PercentageFeatureSet function, while the FindVariableFeatures function is used to identify highly variable genes. Use the ScaleData function to scale all genes and perform a Principal Component Analysis (PCA) reduction. The cells were clustered using the FindNeighbours and FindClusters functions. Subsequently, cell type annotation was conducted utilising the SingleR, CellMarker database and PanglaoDB databases^12^,^13^.

### scPagwas analysis and Bayesian inverse convolution analysis

Trait-related scores (TRS) were calculated for each cell type using the R language package scPagwas in order to assess the role of cell type in relation to neuroblastoma. Furthermore, the correlation between distinct cell types and neuroblastoma was determined by the scPagwas_main function^8^. Furthermore, the R language package DESeq2 was employed for the analysis of differentially expressed genes in neuroblastoma versus control samples. In this study, ganglioneuroblastoma was employed as a control group due to the unavailability of normal tissue. Subsequently, the R language package BayesPrism was employed to conduct the requisite analysis and obtain an estimate of the proportion of each cell type in each sample, that is to say, the Theta values^9^.

### Differential and survival analysis of convolutional cells

The R language packages rstatix, tidyverse and ggsignif were employed for the purpose of analyzing the differences between neuroblastoma and controls for each cell type. Furthermore, we evaluated the prognostic discrepancies between the various cell types in neuroblastoma through Kaplan-Meier analyses, with the cut-off value defined as the median Theta value. *P* < 0.05 was deemed to be statistically significant^14^.

### Association of convolutional cells with clinical features

Furthermore, we conducted a detailed analysis of the differentially expressed trait genes between the various convoluted cellular fractions using the R language package DESeq2. Additionally, we employed the Estimate algorithm to assess the tumor microenvironment (TME) between the different convoluted cell groupings^15^. In this study, all convolutional cells were grouped in accordance with the median Theta value of the individual convolutional cells. Furthermore, the correlation between each convoluted cell and clinical characteristics, including age, gender, risk stratification and clinical stage, was analyzed.

### Analysis of convolutional cells with immune gene sets and immunotherapy

The correlation between convoluted cellular fractions and a total of 17 immune gene sets, including Antigen_Processing_and_Presentation, Chemokines, and TNF_Family_Members, was subjected to further analysis. The response of the high and low convoluted cell groups to immunotherapy was subsequently analyzed using a number of metrics, including Tumor Immune Dysfunction and Exclusion (TIDE) and CD8. Ultimately, we employed the R language package oncoPredict to forecast the drug sensitivity of diverse cell groupings, with the objective of devising more efficacious therapeutic strategies for clinical application^16^.

### Screening of hub genes and analysis of survival, function and relevance

The hub genes of neuroblastoma were identified through the analysis of differentially expressed genes from bulk RNA-seq data. This analysis involved the intersection of trait-related genes with marker genes in convoluted cellular fractions. We further used the Kyoto Encyclopedia of Genes and Genomes (KEGG) set obtained from MSigDB for KEGG enrichment analyses of differential genes, and the DOSE package for Gene Ontology (GO) enrichment analyses of differential genes obtained from screening^17^,^18^. Furthermore, we conducted a Gene Set Enrichment Analysis (GSEA) analysis on the hub genes identified through screening to ascertain their underlying biological functions^19^. The prognosis of the hub genes was additionally evaluated using Kaplan-Meier analysis, and the association of the hub genes with convoluted cellular fractions was ultimately analyzed using correlation analysis.

### SMR validates hub genes

Furthermore, the hub genes in neuroblastoma were identified through the utilisation of SMR software in conjunction with GWAS summary data^20^.

### RNA extraction and quantitative real-time polymerase chain reaction (qRT-PCR) analysis

In the present study, a total of 74 clinical neuroblastoma RNA specimens and 8 paied para-tumor RNA specimens were obtained from the Cancer Biobank of Tianjin Medical University Cancer Institute and Hospital (Tianjin, China) between 2008 and 2021. The study was approved by the Research Ethics Review Committee of Tianjin Medical University Cancer Institute and Hospital. Informed consent was obtained from all participants prior to the commencement of the study (E20210664).

RNA samples were reverse transcribed using PrimeScript RT Master Mix (Takara Bio). qRT-PCR was performed using SYBR Green Premix SupTaq HS (Accurate Biology). Glyceraldehyde 3-phosphate dehydrogenase (GAPDH) expression levels were used as a standard reference. The entire qRT-PCR process was performed using an Applied Biosystems QuantStudio 5 Real-Time PCR System. The 2^-ΔΔCt^ method was used to analyze the relative expression levels of mRNAs. The primers used are listed in Supplementary Table (Supplementary Table 1).

### Statistical analysis

All analytical techniques, data visualization and statistical analyses employed in this study were conducted using R software (version 4.4.1) and the R package integrated within the software. All statistical p-values were two-sided, and *p* < 0.05 was considered statistically significant.

## Results

### Identification of neuroblastoma cell subtypes

The single-cell data obtained from GEO (https://www.ncbi.nlm.nih.gov/geo/query/acc.cgi?acc=GSE218450) was initially filtered using Seurat 4.0, resulting in the removal of substandard cells and the subsequent availability of cells for further analysis (Fig. 1A). Furthermore, the FindVariableFeatures function was employed to identify genes exhibiting high variability, resulting in the identification of 2000 genes that met this criterion (Fig. 1B). We scaled all genes using the ScaleData function and performed Principal Component Analysis (PCA). Significant cell separation was observed in the individual neuroblastoma samples (Fig. S1A). In the PCA, the top 20 principal components (PCs) were selected for further analysis at a significance level of *P* < 0.05 (Fig. 1E, F). The Uniform Manifold Approximation and Projection (UMAP) and t-distributed Stochastic Neighbor Embedding (t-SNE) algorithms were employed to facilitate the further categorisation of the core cells of the neuroblastoma into 21 distinct cell clusters (Fig. S1B,C). The marker genes of individual cell clusters were annotated using the singleR, CellMarker and PanglaoDB databases. The core cells of neuroblastoma were further annotated as IL7R/CD4^+^ T cells, Neurons, FYB/CD4^+^ T cells, NK cells, Regulatory T cells, IGKC/B cells, Endothelial cells and M2 macrophages, resulting in a total of eight cell types (Fig. 1C,D). Furthermore, our findings revealed that the ratio of FYB/CD4^+^ T cells and IL7R/CD4^+^ T cells was elevated in MYCN-amplified neuroblastoma (Fig. S1D). The findings indicate the existence of distinct cellular populations within neuroblastoma, which may be associated with the intratumoural heterogeneity observed in these neoplasms.

### scPagwas analysis and Bayesian inverse convolution analysis

The annotated single-cell data was analyzed using the scPagwas algorithm, which revealed that FYB/CD4+ T cells exhibited the highest Trait-related scores (TRS) and were significantly different from the other cell types (Fig. 2A). Furthermore, the application of this algorithm enabled the identification of trait genes, including RPLP1, PRKCA and HLA-B, which are associated with the occurrence of neuroblastoma (Fig. S2A). Furthermore, we obtained bulk RNA-seq data of neuroblastoma from the TCGA database (https://www.cancer.gov/ccg/research/genome-sequencing/tcga) and conducted a differential gene expression analysis between neuroblastoma and control samples. The results demonstrate that genes such as GFAP, PLEKHS1, etc. are upregulated and CALB1 and LHX9, etc. are downregulated in neuroblastoma tissues (Fig. S2B). Bayesian inverse convolution calculations were performed based on the scPagwas algorithm, resulting in the calculation of Theta values for each cell type in each patient (Fig. S2C). Kaplan-Meier analyses were performed on the basis of the Theta values of individual cells in conjunction with clinical data. The results demonstrated a statistically significant survival discrepancy between FYB/CD4+ T cells and IL7R/CD4+ T cells in neuroblastoma (Fig. 2B,C). Similarly, the Theta values of various types of convoluted cellular fractions in neuroblastoma cells were subjected to analysis. Significant differences were observed between FYB/CD4+ T cells, IL7R/CD4+ T cells, NK cells and M2 macrophages in control versus neuroblastoma tissues (Fig. 2D). In light of the aforementioned evidence, it can be posited that the FYB/CD4+ T cell plays a more significant role in the development, advancement, and prognosis of neuroblastoma than other cells. Consequently, further investigation was conducted on the FYB/CD4+ T cell.

### Tumor microenvironment (TME) and clinical features of FYB/CD4+ T cells

We classified FYB/CD4+ T cells into high and low groups based on convoluted cell Theta values. The results demonstrated notable discrepancies in the estimated scores of FYB/CD4+ T cells across distinct subgroups, along with considerable variations in the composition and tumor purity of FYB/CD4+ T cells within different subgroups in comparison to other cell types (Fig. 3A). Specifically, low levels of FYB/CD4+ T cells were associated with distinct tumor microenvironment alterations: decreased abundances of Neurons and IGKC/B cells, alongside enriched populations of Regulatory T cells, Endothelial cells, and M2 macrophages (Fig. 3A). These patients also presented with significantly higher Stromal, Immune, and ESTIMATE scores, yet lower tumor purity, compared to their high-FYB/CD4+ T cell counterparts (Fig. 3A). In addition, we further found that compared to the low Theta value group, the high Theta group had higher expression of HRG, APOA2, and ALB, etc., and lower expression of CALCA, HSD3B2, and STAR, etc. (Fig. 3A,B). Furthermore, the relationship between different subgroups of FYB/CD4+ T cells and the clinical features of neuroblastoma was subjected to additional analysis. The findings demonstrate that discrepancies in FYB/CD4+ T cell occupancy are associated with clinical staging and risk stratification of neuroblastoma, irrespective of age and gender (Fig. 3C-F). Our study demonstrated that FYB/CD4+ T cells were differently represented in neuroblastoma, correlating with TME, immune infiltration and clinical features of neuroblastoma. The heterogeneity in neuroblastomas was further confirmed.

### FYB/CD4+ T cell associations with various immune gene sets

The GSEA database was queried to obtain a number of immune gene sets, which were then subjected to analysis to determine the association between different FYB/CD4+ T cell ratio groupings and various immune gene sets. Our results showed that the FYB/CD4+ T cell ratio was associated with the Cytokines gene set (Fig. 4), the Cytokine Receptors gene set (Fig. S5C), the TCR signaling Pathway gene set (Fig. 5), and the Antimicrobials gene set (Fig. S3), BCR Signaling Pathway gene set (Fig. S4B) and almost all genes in the Chemokines gene set (Fig. S4C) are associated. Additionally, our findings indicated that distinct FYB/CD4+ T cell ratio categorizations were linked to the expression of CD8A, CD8B, CREB1, CTSB, and HSPA1B in the Antigen Processing and Presentation genes (Fig. S4A). Furthermore, our findings suggest a correlation between the ratio of FYB/CD4+ T cells and gene expression in the Chemokine Receptors and Natural Killer Cell Cytotoxicity gene sets (Fig. S5A,B). The present study demonstrates that disparate ratios of FYB/CD4+ T cells in neuroblastoma are frequently correlated with the expression of multiple immune genes. This may be associated with the regulation of immune genes.

### Immunotherapy analysis of FYB/CD4+ T cells

The application of immunotherapy has been extended to a range of tumors, including neuroblastoma, with associated improved patient outcomes^21^. We analyzed the correlation between different FYB/CD4+ T cell ratios and immunotherapy response. Our results showed higher values of Cancer-Associated Fibroblasts (CAF), Dysfunction and TIDE scores in the group with lower FYB/CD4+ T cell ratio (Fig. 6A,D,G). We also found that lower ratios of FYB/CD4+ T cells were associated with lower values of CD8, Cytotoxic T Lymphocyte (CTL), Myeloid-Derived Suppressor Cell (MDSC) and TAM M2 scores (Fig. 6B,C,E,F). The findings of our study indicate that the response to immunotherapy is dependent on the ratio of FYB/CD4+ T cells in the neuroblastoma. The analysis of FYB/CD4+ T cells can assist clinicians in selecting the most appropriate immunotherapy regimen and improving patient survival rates.

### Drug sensitivity analysis of FYB/CD4+ T cells

The half maximal inhibitory concentration (IC50) is an indicator of the response rate of a chemotherapeutic drug to tumor cells. The IC50 value can be used as a measure of the drug's ability to induce apoptosis, whereby a stronger induction is reflected in a lower value. In the context of oncological studies, the IC50 value is typically indicative of the capacity of tumor cells to withstand the effects of pharmacological agents^22^. The sensitivities of chemotherapeutic drugs were analyzed in relation to different FYB/CD4+ T cell ratios. The findings revealed that the sensitivity of distinct FYB/CD4+ T cell subgroups to the majority of chemotherapeutic agents exhibited notable disparities. Additionally, a low proportion of FYB/CD4+ T cells demonstrated a lack of sensitivity to the majority of chemotherapeutic agents (Fig. 7).

We further selected some of the commonly used chemotherapeutic agents in neuroblastoma from the literature to further demonstrate the IC50 differences between different chemotherapeutic agents^23^. The findings indicated that the decline in the ratio of FYB/CD4+ T cells was linked to diminished drug sensitivity to cisplatin, cyclophosphamide, irinotecan and vincristine (Fig. S6A-D). The findings of this study indicate that the ratio of FYB/CD4+ T cells may influence the sensitivity of chemotherapeutic agents, thereby affecting the therapeutic effect and resulting in further survival differences.

### Screening and functional analysis of hub genes

We identified 18 hub genes in neuroblastoma based on differentially expressed genes in the bulk RNA-seq (p.adj < 0.01, ∣log2FC∣ > 2), genes in the Marker of FYB/CD4+ T cells, and the top 500 genes in Pearson correlation coefficients (PPC) (Fig. 8A). The 18 genes were subjected to analysis for GO, KEGG and DO enrichment. The results demonstrated that the hub genes were markedly enriched in the intracellular receptor signaling pathway, the TNF signaling pathway, and in both nervous system and adrenal gland cancers (Fig. 8B,C). A Kaplan-Meier analysis was conducted on the 18 hub genes. The results demonstrated that ten hub genes, including HBA2 and RGS1, may influence the prognosis of neuroblastoma (Fig. 8D, E; Fig. S7). To further identify the hub genes of neuroblastoma, we selected genes that were highly expressed in neuroblastoma with poor prognosis and those that were lowly expressed in neuroblastoma with good prognosis as the hub genes of neuroblastoma. The results of our analysis indicate that HBA2 and RGS1 may be considered as potential hub genes. A GSEA enrichment analysis was conducted on these two genes. Our results showed that HBA2 was significantly enriched in Cholesterol metabolism, Neuroactive ligand-receptor interaction and Steroid hormone biosynthesis (Fig. 8F). In addition, RGS1 was significantly enriched in Antigen processing and presentation and Butanoate metabolism (Fig. 8G). Our study demonstrates the important role of HBA2 and RGS1 in the occurrence and development of neuroblastoma.

### Correlation between hub genes and individual convolutional cells

We further analyzed the correlation between the expression of HBA2 and RGS1 and the proportion of each cell type. Our results showed that RGS1 expression was positively correlated with the proportions of Endothelial cells (Fig. 9A), M2 macrophages (Fig. 9E), NK cells (Fig. 9G) and Regulatory T cells (Fig. 9H) and negatively correlated with the proportions of FYB/CD4+ T cells (Fig. 9B) and Neurons (Fig. 9F). In addition, there was no significant correlation between RGS1 expression and cell proportions of IGKC/B cells (Fig. 9C) and IL7R/CD4+ T cells (Fig. 9D). In HBA2, although similar results were also seen, the relationship between HBA2 expression and the cellular ratio of IL7R/CD4+ T cells was more significant (Fig. 10A-H). Our study shows that the expression of RGS1 and HBA2 correlates with the composition of cell ratios, which may be due to the regulation of multiple cells including T cells by hub genes.

### SMR analysis to validate hub genes

The hub genes were additionally validated in neuroblastoma through SMR analysis. The results demonstrated that RGS1, EPHA2 and ARHGEF19 may be associated with the occurrence of neuroblastoma (Fig. 11). The results of our study indicate that RGS1 may serve as a novel biological signature molecule in the context of neuroblastoma.

### RGS1 is overexpressed in neuroblastoma and is clinically relevant

The relative RNA expression level of RGS1 in each sample was determined by qRT-PCR. We used qRT-PCR to analyze RGS1 expression in tumor tissues and their para-tumor tissues in 8 paired neuroblastoma samples. RGS1 expression was significantly upregulated in neuroblastoma samples compared with the corresponding adjacent benign samples (Fig. S8A). We subsequently measured the expression levels of RGS1 in 74 clinical neuroblastoma samples, and the results showed that the expression of RGS1 was markedly higher in patients with stage 4 tumors compared with in patients with stage 1 or stage 2 and 3 tumors (Fig. S8B). Furthermore, we performed a pan-cancer analysis using the TCGA database to evaluate RGS1 expression in other tumor types, and the results showed that RGS1 was highly expressed in most tumors (Fig. S8C), consistent with our findings.

## Discussion

Advances in single-cell sequencing permit the study of the variability of individual cells and the expression of complex genes, thereby revealing the heterogeneity among different cells^24^. Single-cell studies have been extensively employed in tumor research as an efficacious instrument for investigating the TME^25^,^26^. At present, an increasing number of studies of neuroblastoma are employing single-cell analysis. Dong *et al*. and Jansky *et al*. have utilized this approach to confirm the cellular state and developmental trajectory of neuroblastoma^6^,^27^. Baryawno *et al*. and Costa *et al*. conducted further analysis of the tumor immune microenvironment (TIME) in neuroblastoma using single-cell data, thereby providing new directions for neuroblastoma research and treatment^28^,^29^. However, past studies have almost always examined and analyzed single-cell data separately from bulk RNA-seq data, and have not normalized the two into the same kind of data. Fortunately, the advent of scPagwas and Bayesian Inverse Convolution has brought new opportunities^8^,^9^. In the neuroblastoma study, we performed the first comprehensive analysis of scRNA-seq and bulk RNA-seq. In addition, we combined GWAS and SMR analyses to further comprehensively analyze neuroblastoma.

Our study identified eight cell types in neuroblastoma, reflecting the heterogeneity of neuroblastoma tumors. The highest levels of neuronal cells were identified in the tumor tissue, which is consistent with the origin of neuroblastomas^23^. Furthermore, our findings revealed a notable elevation in the proportion of CD4^+^ T cells among patients with MYCN amplification. It is noteworthy that MYCN amplification is regarded as the most conventional marker of high-risk neuroblastoma^30^. CD4^+^ T cells, traditionally regarded as the central coordinators of adaptive immunity, play a pivotal role in initiating antitumor immune responses by activating cytotoxic T cells, B cells, and macrophages^31^. However, within the unique immune microenvironment of neuroblastoma, their function demonstrates a remarkable duality, as their antitumor potential is often suppressed by potent immunosuppressive mechanisms. Recent studies have revealed that tumors can actively "hijack" specific CD4^+^ T cell subsets, converting them into accomplices for tumor progression. For instance, Van der Velde *et al*. discovered that a non-conventional subset of CD4^+^ T cells can form functional units with myeloid cells expressing arginase-1 within the tumor parenchyma. Together, they establish an immunosuppressive niche that supports tumor growth, the presence of which is even essential for tumor formation^32^. Nevertheless, the therapeutic potential of CD4^+^ T cells remains undiminished. Through cutting-edge immune engineering strategies, such as genetically modifying them into GD2- or GPC2-targeting CAR-T cells and combining them with synthetic extracellular vesicles that enhance T cell persistence, their potent direct and indirect cytotoxic capabilities can be successfully "unlocked." This approach demonstrates considerable promise for reversing immunosuppression and achieving effective tumor eradication^33^. Therefore, CD4^+^ T cells in neuroblastoma resemble a "functional switch"; whether they ultimately promote or combat the tumor is profoundly determined by their intricate interactions with the tumor microenvironment. Future therapeutic breakthroughs will likely depend on how precisely this "switch" can be regulated.

We further categorized CD4^+^ T cells and identified two new classes of CD4^+^ T cells, FYB/CD4^+^ T cells and IL7R/CD4^+^ T cells. *FYB* can function as an interface protein for signaling among T cells^34^. In addition *IL7R* plays an important role in B cell maturation^35^. We believe that FYB is of interest in studies of neuroblastoma, especially regarding CD4^+^ T cells.

The scPagwas algorithm revealed a strong correlation between FYB/CD4^+^ T cells and the neuroblastoma trait, as well as a significant correlation between these cells and other cell populations. Furthermore, our findings revealed a reduction in the ratio of FYB/CD4^+^ T cells in neuroblastoma, which was associated with an increased risk of worse clinical grading, risk stratification, and prognosis. The study by Tang *et al*. reported that depletion of CD4^+^ T cells was associated with poor prognosis in neuroblastoma^36^. This is in general agreement with our study. We suggest that FYB/CD4^+^ T cells may have an important role in the development as well as prognosis of neuroblastoma.

Immunotherapy and chemotherapy have been widely used in the treatment of neuroblastoma^37^. We analyzed the TIME profile of different FYB/CD4^+^ T cells ratios. Our findings revealed that the cohort exhibiting a low ratio of FYB/CD4^+^ T cells demonstrated inferior immune infiltration and a paucity of pure tumors. Lower tumor purity often predicts a higher degree of malignancy^15^,^38^,^39^. Furthermore, we conducted an analysis of FYB/CD4^+^ T cells in conjunction with immune gene sets. A reduction in the ratio of FYB/CD4^+^ T cells frequently preceded an association with low gene expression levels associated with various immune gene sets. Further investigation revealed that lower FYB/CD4^+^ T cells exhibited lower CD8 expression and higher scores on the TIDE scale. The most important function of the CD8 molecule is to participate in the transduction process of activation signals generated by TCR recognition of antigens and to enhance the interaction between T cells and antigen-presenting cells (APCs) or target cells^40^-^42^. In addition, higher TIDE scores tended to predict poorer immune checkpoint inhibition therapy (ICI) efficacy^43^,^44^. Furthermore, we investigated the chemotherapeutic drug sensitivity of FYB/CD4^+^ T cells and observed that a low percentage of FYB/CD4^+^ T cells exhibited generally poor drug sensitivity, particularly in response to several commonly utilized chemotherapeutic agents in neuroblastoma. These may be related to the poor staging and prognosis of low FYB/CD4^+^ T cells. Therefore, FYB/CD4^+^ T cells are of interest in immunotherapy and chemotherapy for neuroblastoma.

We conducted a comprehensive analysis integrating GWAS, scRNA-seq, and bulk RNA-seq data to identify two key hub genes associated with neuroblastoma, specifically *RGS1* and *HBA2*. Among them, *HBA2* was significantly enriched in Cholesterol metabolism, Neuroactive ligand-receptor interaction and Steroid hormone biosynthesis, while *RGS1* was significantly enriched in Antigen processing and presentation. Both have a strong correlation with various cell populations, especially immune cell populations. It has been demonstrated that cholesterol metabolism and antigen processing and presentation are significant factors influencing the onset, progression, and prognosis of neuroblastoma^45^-^47^. Finally, we conducted an additional verification of the neuroblastoma core genes through SMR analysis, which revealed that *RGS1* may serve as a pivotal hub gene in neuroblastoma. Accordingly, further investigation of *RGS1* is warranted in subsequent studies on neuroblastoma.

It should be noted that the present study is not without limitations. Firstly, our study was based almost entirely on public databases, which require further validation in subsequent studies. Secondly, we identified some updated datasets on neuroblastoma while conducting our study, which we can validate using more updated datasets in subsequent studies. Similarly, our study was deficient in the inclusion of large prospective cohort studies, which should be addressed in subsequent studies.

In this study, we employed the scPagwas algorithm in conjunction with Bayesian inverse convolution to synthesize neuroblastoma for the first time. A comprehensive analysis of GWAS data, scRNA-seq, and bulk RNA-seq data of neuroblastoma was performed, resulting in the identification of a new class of cell populations, namely FYB/CD4^+^ T cells, that may affect the occurrence, progression, and survival of neuroblastoma. Based on the ratio of FYB/CD4^+^ T cells, an effective assessment of the immunotherapeutic response of neuroblastoma can be conducted, thereby assisting clinical clinicians in the selection of new chemotherapeutic agents. Ultimately, our findings suggest that *RGS1* may serve as a potential hub gene in neuroblastoma, as evidenced by the results of the SMR analysis and clinical specimen validation.

## Supplementary Material

Supplementary figures and tables.

## Figures and Tables

**Figure 1:**
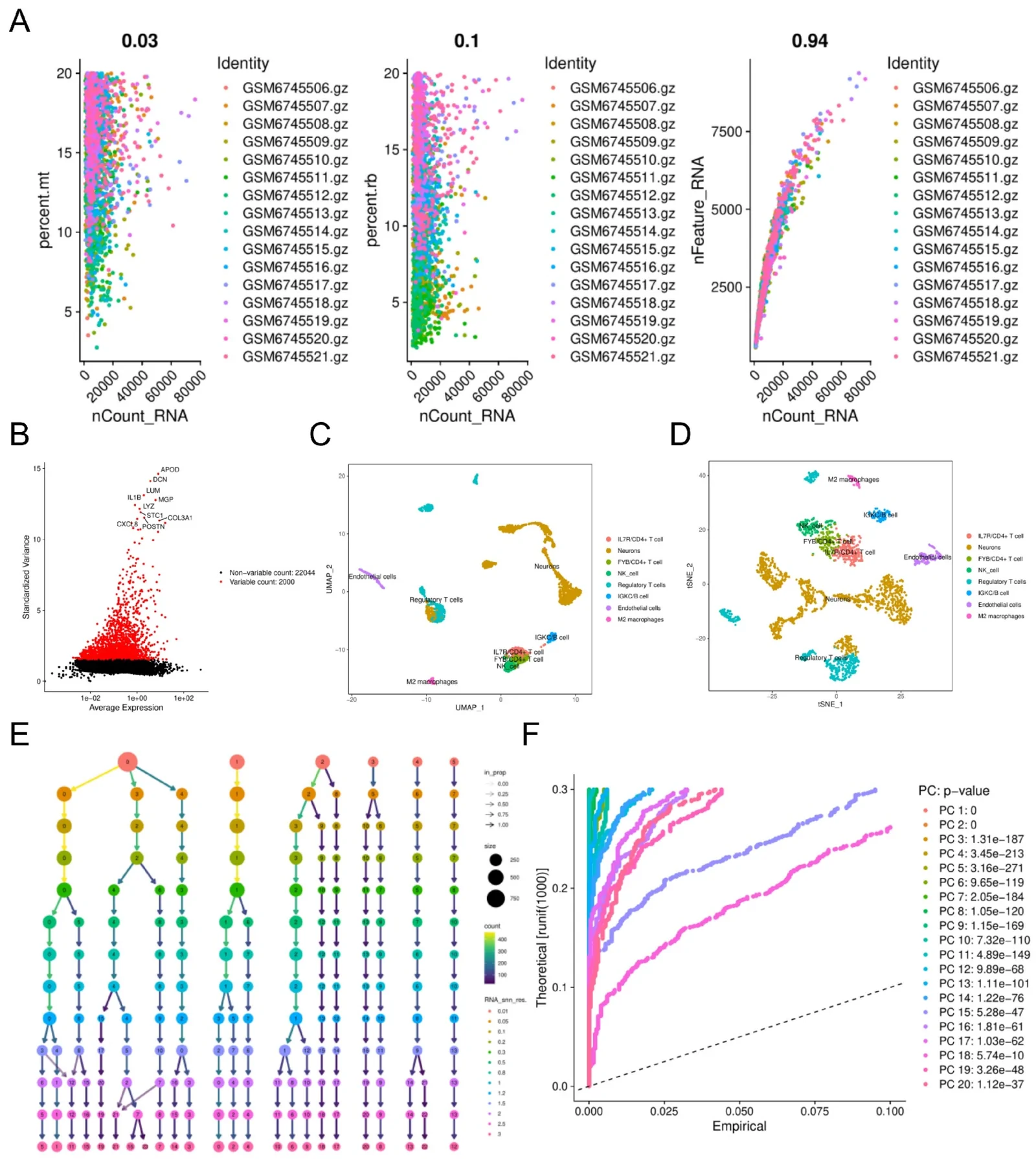
Filtering and Annotation of scRNA-Seq Data. A: The scRNA-seq data were filtered to filter out ineligible cells for subsequent analysis; B: the variance plot shows the variation of gene expression in all cells of neuroblastoma. The red dots represent highly variable genes and the black dots represent non-variable genes; C-D: 8 cell clusters were annotated based on the expression of marker genes; E-F: PCA identified the top 20 PCs at P < 0.05.

**Figure 2:**
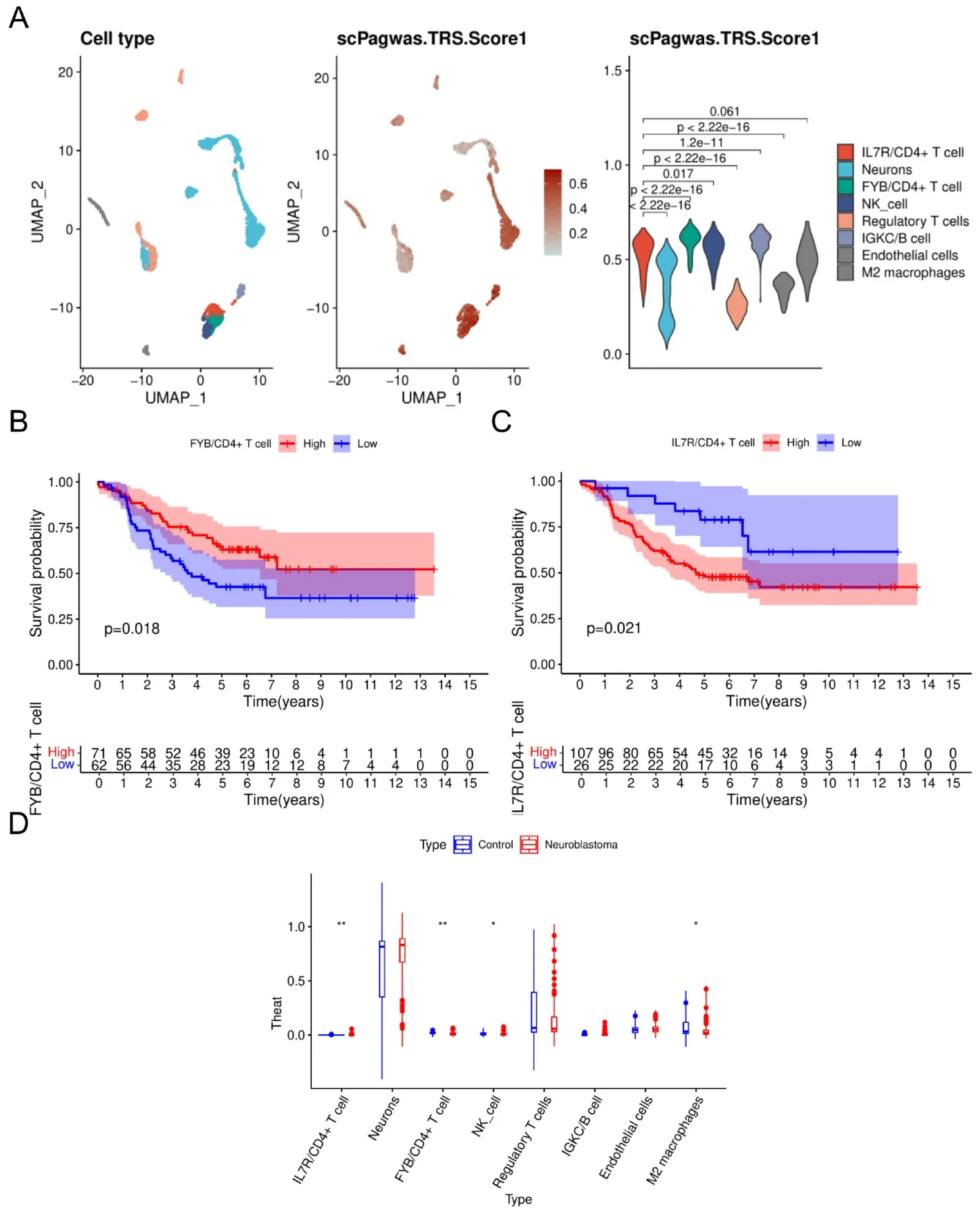
scPagwas analysis and Bayesian inverse convolution analysis. A: Individual cell TRS scores based on the scPagwas algorithm; B: K-M curve of FYB/CD4+ T cell; C: K-M curve of IL7R/CD4+ T cell; D: Differences between single cells in control and neuroblastoma. *P < 0.05; **P < 0.01; ***P < 0.001.

**Figure 3:**
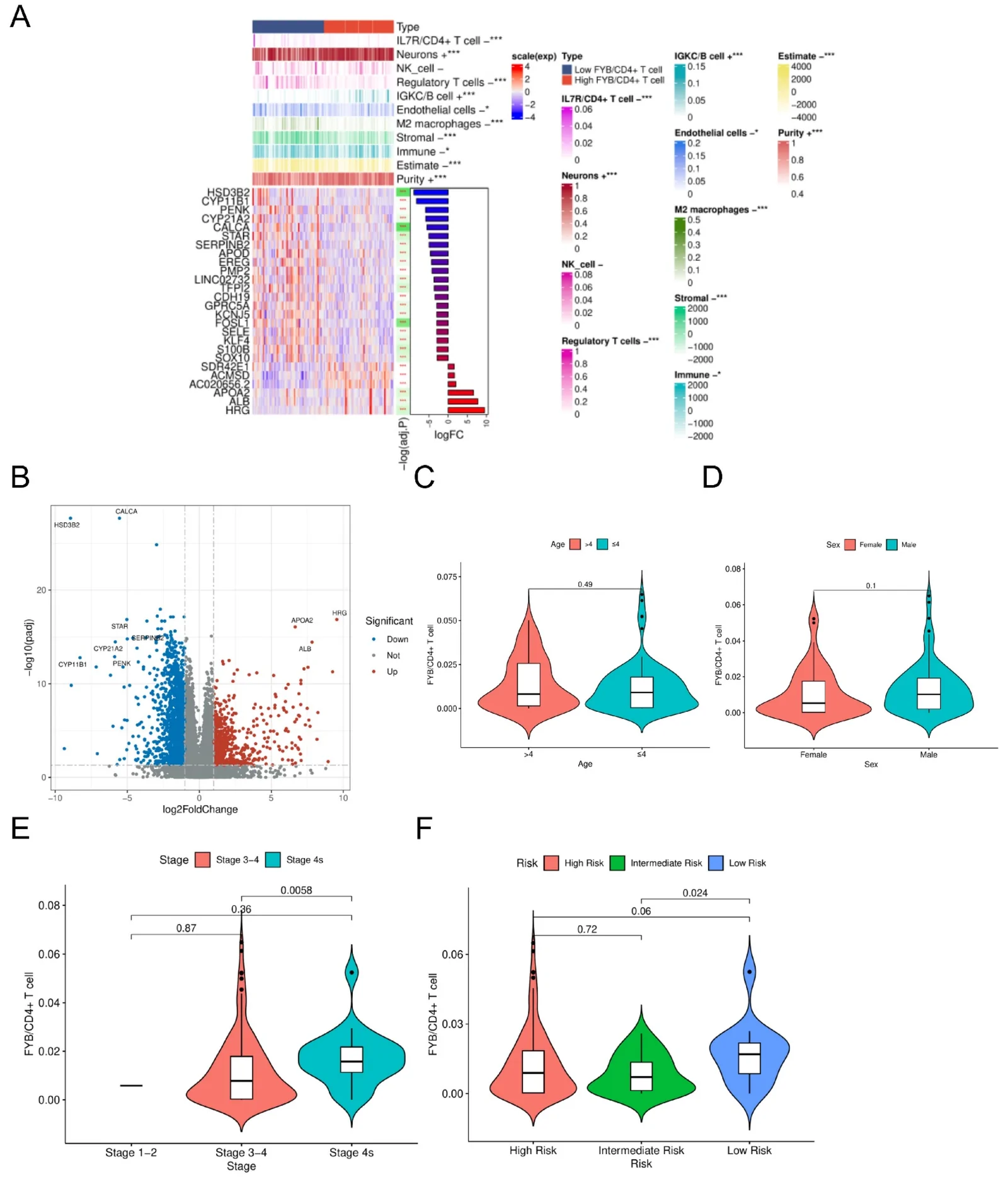
TME and clinical characteristics of FYB/CD4+ T cells A: TME differences in FYB/CD4+ T cells; B: Differential Maker genes in FYB/CD4+ T cells versus other convoluted cellular fractions; C: Association of FYB/CD4+ T cells with age; D: Association of FYB/CD4+ T cells with sex; E: Association of FYB/CD4+ T cells with stage of disease; F: Association of FYB/CD4+ T cells with risk of disease; *P < 0.05; **P < 0.01;***P < 0.001.

**Figure 4:**
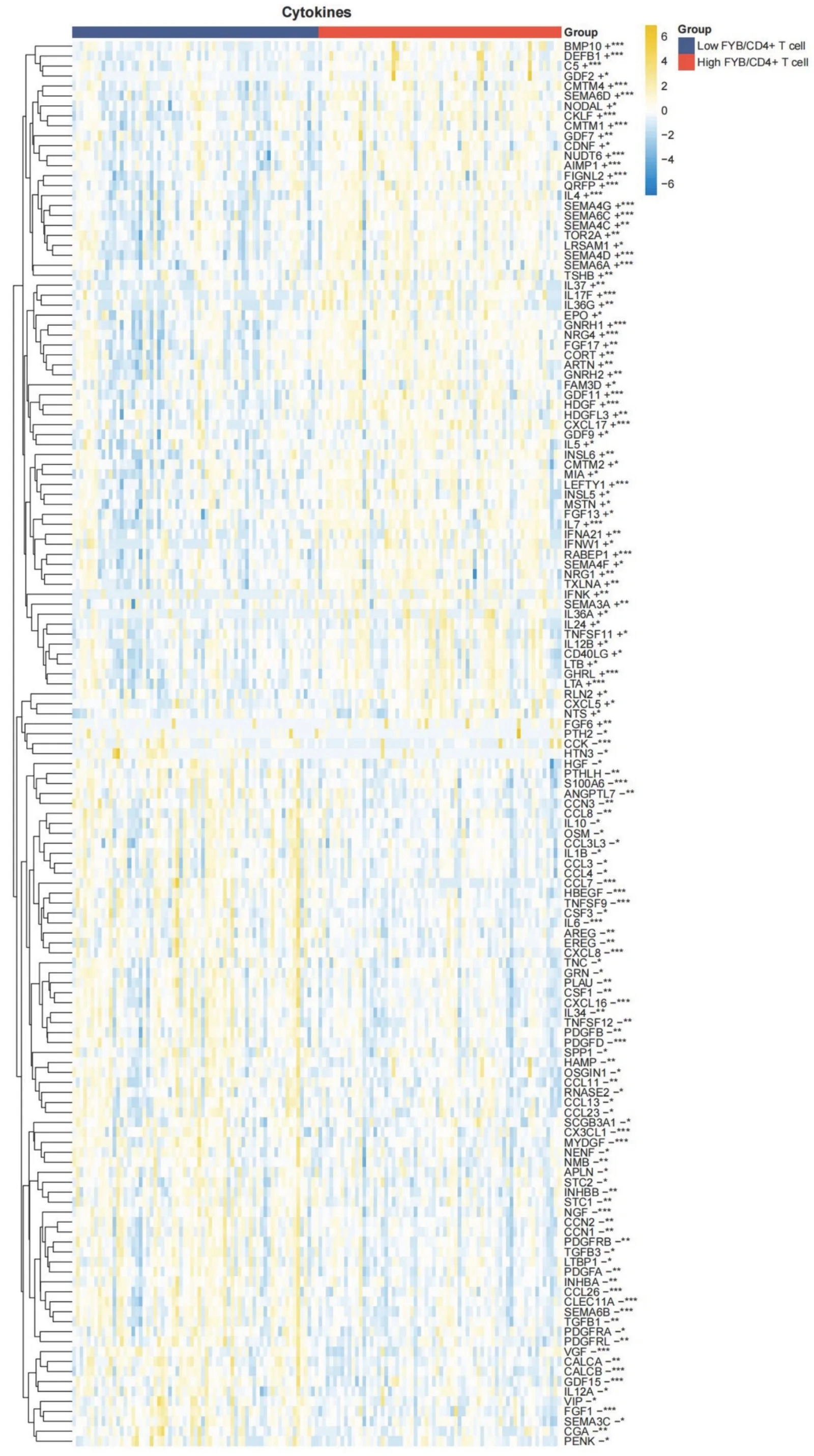
FYB/CD4+ T cell with Cytokines gene set, *P < 0.05; **P < 0.01; ***P < 0.001.

**Figure 5:**
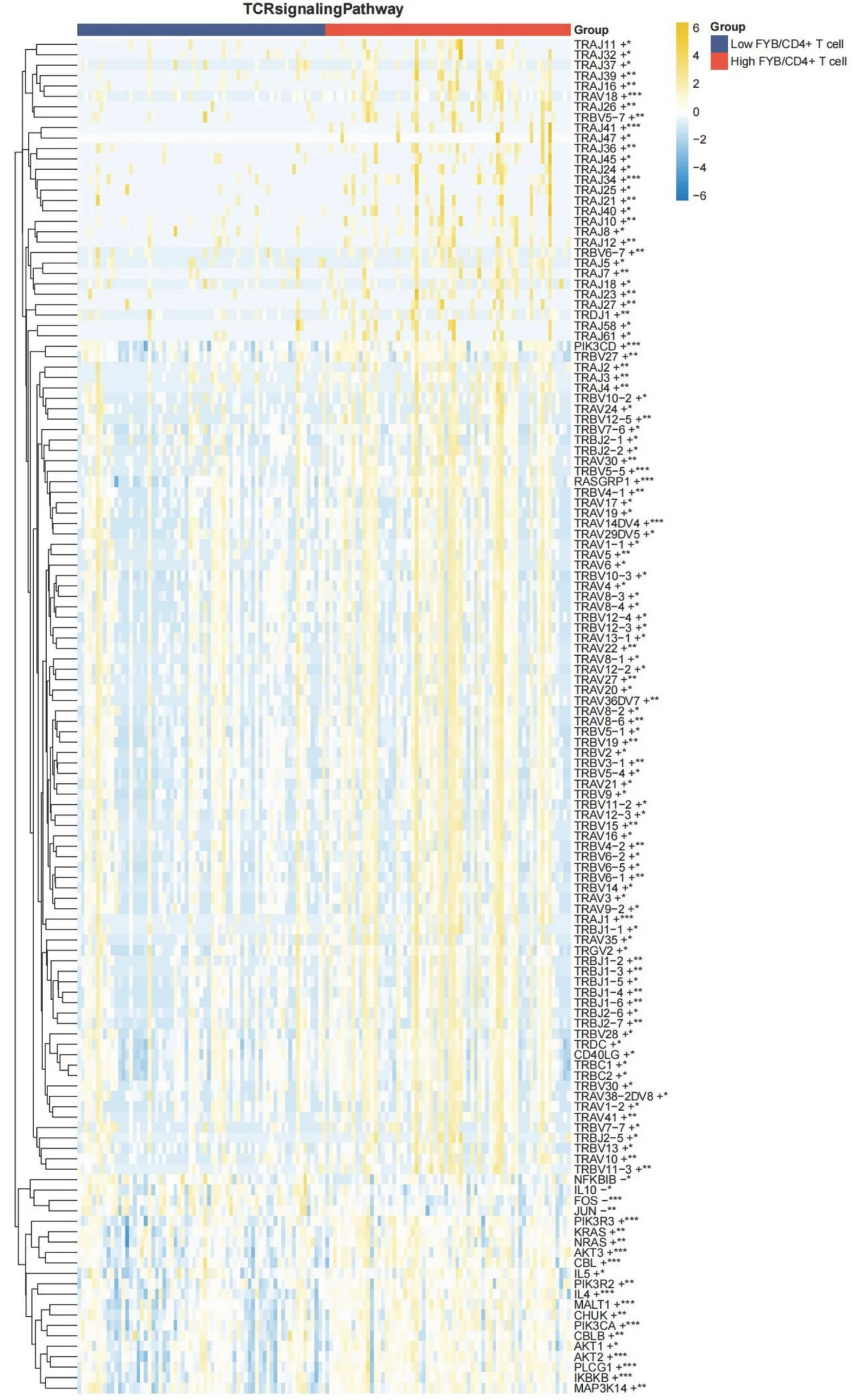
FYB/CD4+ T cell with TCR signaling Pathway gene set, *P < 0.05; **P < 0.01; ***P < 0.001.

**Figure 6:**
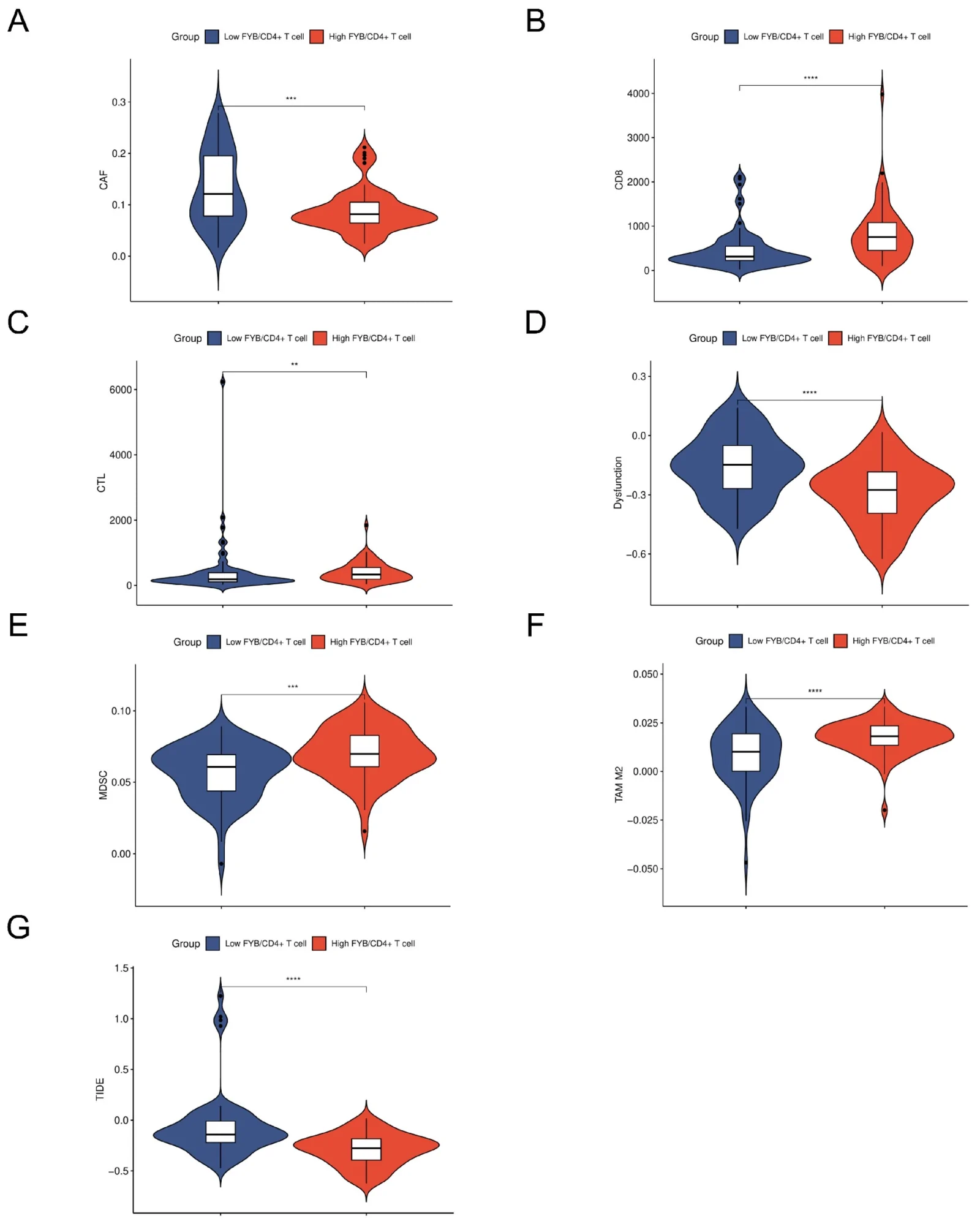
Immunotherapy Analysis of FYB/CD4+ T Cells. A: Association of FYB/CD4+ T cell with CAF; B: Association of FYB/CD4+ T cells with CD8; C: Association of FYB/CD4+ T cell with CTL; D: Association of FYB/CD4+ T cells with Dysfunction; E: Association of FYB/CD4+ T cell with MDSC; F: Association of FYB/CD4+ T cells with TAM M2; G: Association of FYB/CD4+ T cell with TIDE. *P < 0.05; **P < 0.01; ***P < 0.001; ****P < 0.0001.

**Figure 7:**
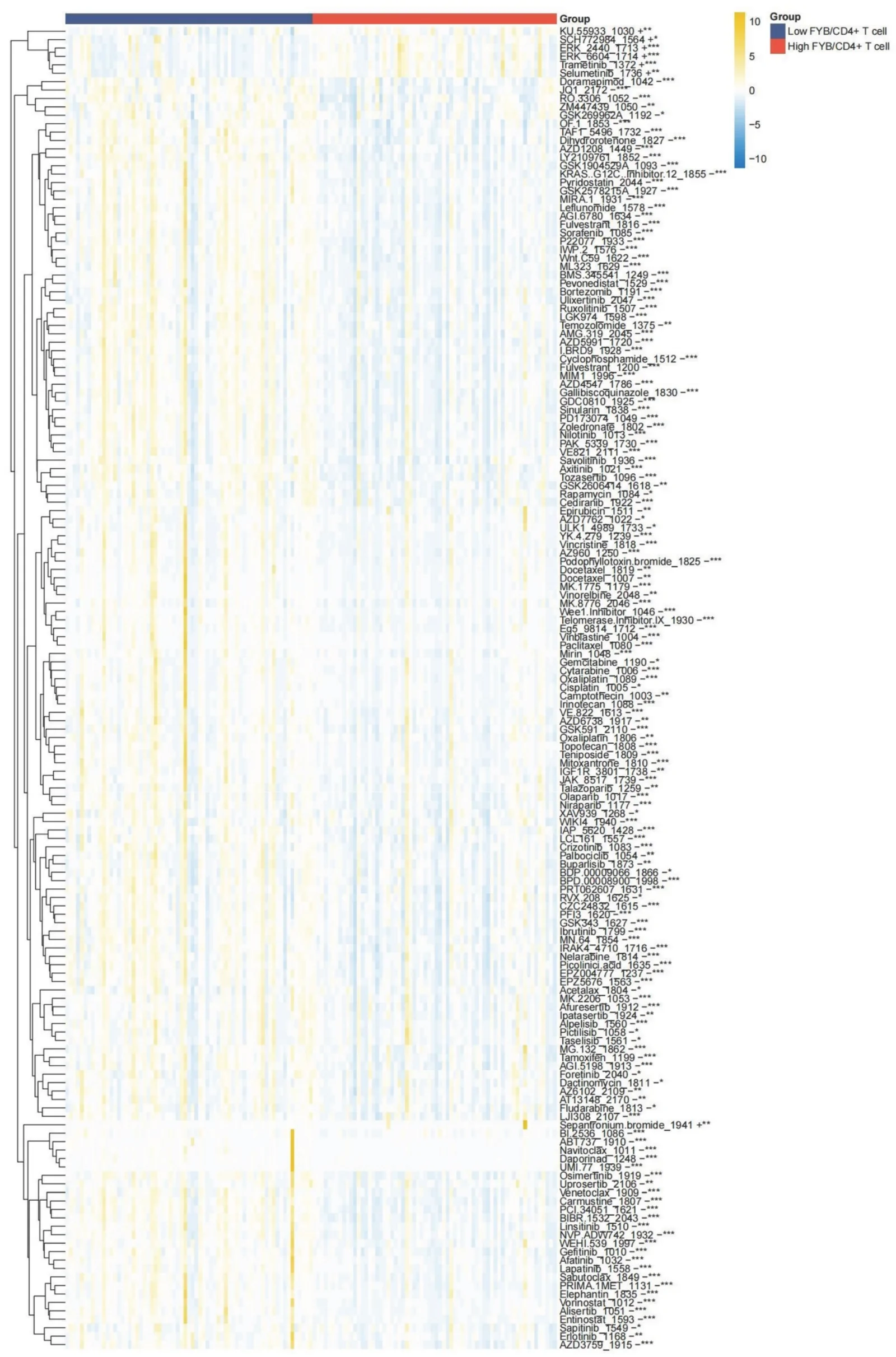
Drug sensitivity analysis, *P < 0.05; **P < 0.01; ***P < 0.001.

**Figure 8:**
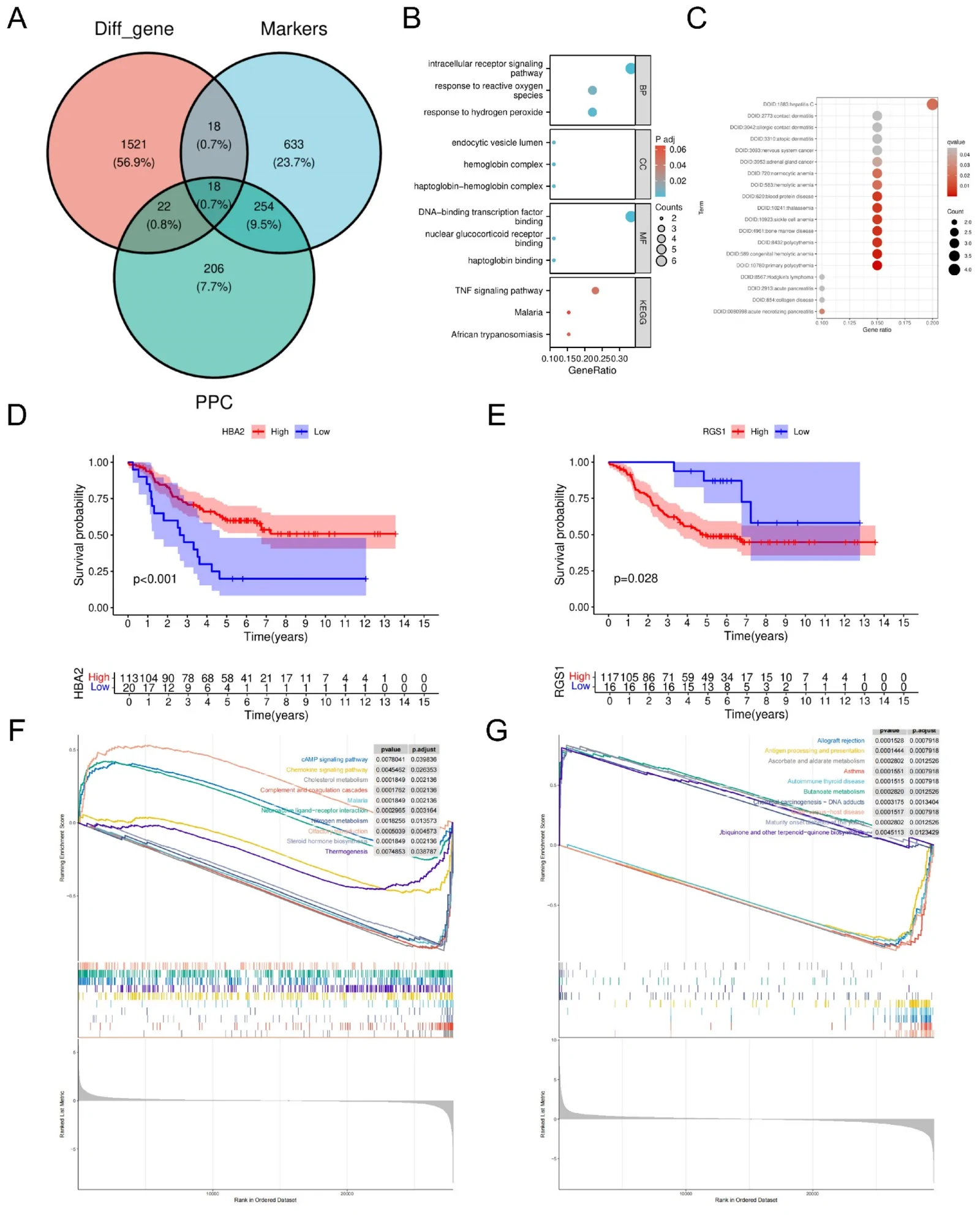
Identification of hub genes. A: Hub gene screening; B: GO and KEGG analysis; C: DO analysis; D: K-M curve of HBA2; E: K-M curve of *RGS1*; F: GSEA analysis of HBA2; G: GSEA analysis of *RGS1*.

**Figure 9:**
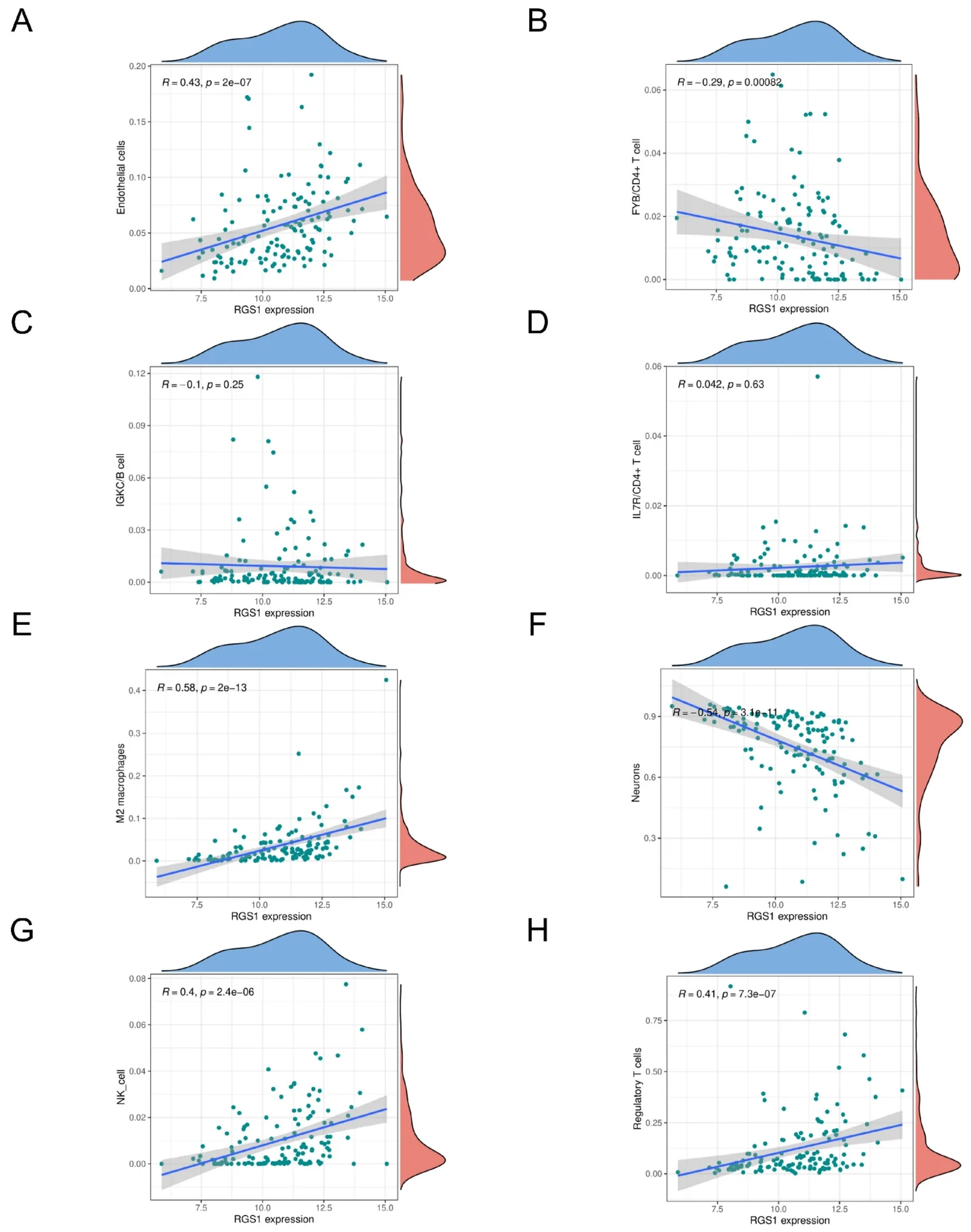
Correlation of RGS1 with single convolutional cells. A: Correlation of RGS1 expression with Endothelial cells; B: Correlation of RGS1 expression with FYB/CD4+ T cells; C: Correlation of RGS1 expression with IGKC/B cell; D: Correlation of RGS1 expression with IL7R/CD4+ T cells; E: Correlation of RGS1 expression with M2 macrophages; F: Correlation of RGS1 expression with Neurons; G: Correlation of RGS1 expression with NK cell; H: Correlation of RGS1 expression with Regulatory T cells.

**Figure 10:**
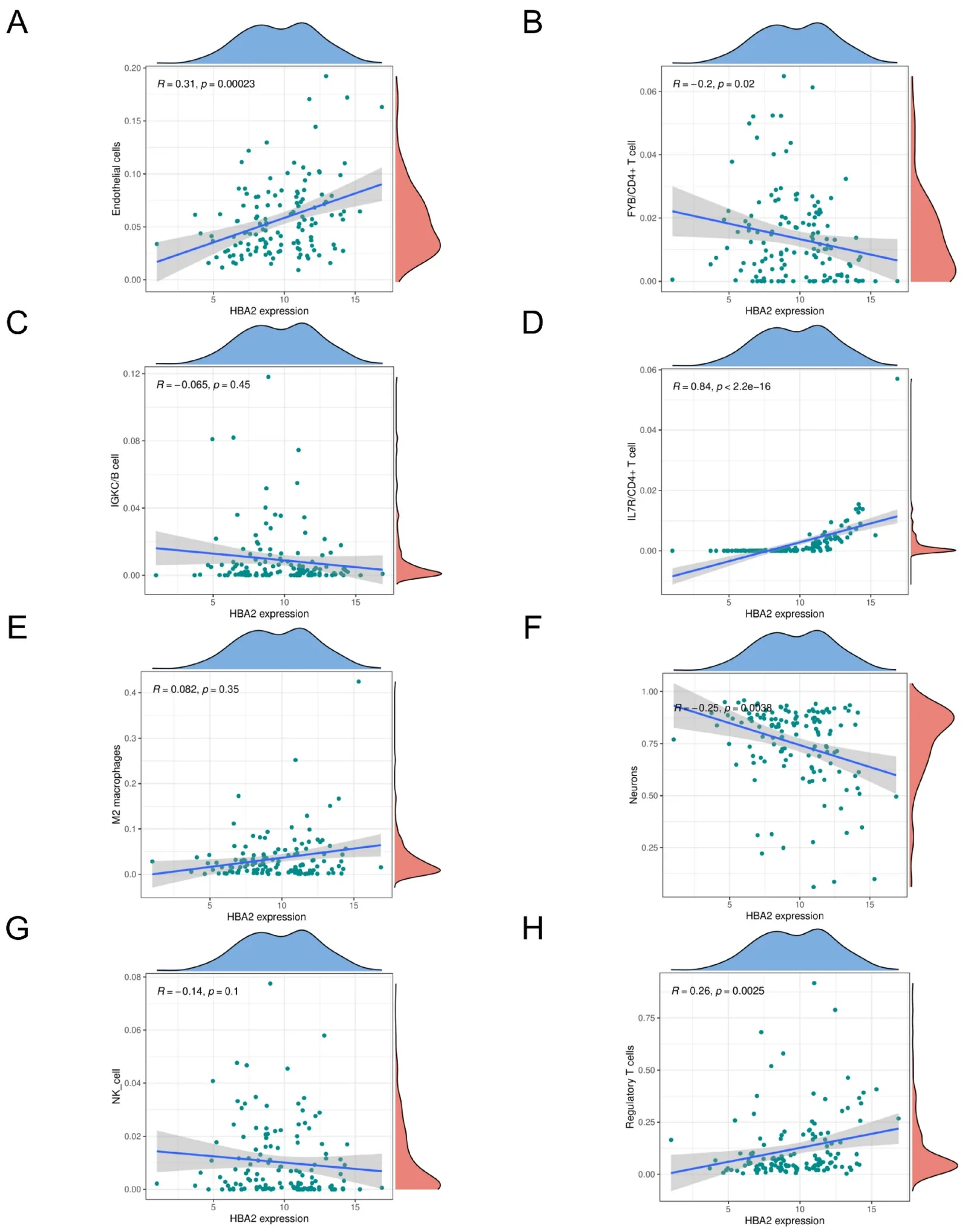
Correlation of HBA2 with single convolutional cells. A: Correlation of HBA2 expression with Endothelial cells; B: Correlation of HBA2 expression with FYB/CD4+ T cells; C: Correlation of HBA2 expression with IGKC/B cell; D: Correlation of HBA2 expression with IL7R/CD4+ T cells; E: Correlation of HBA2 expression with M2 macrophages; F: Correlation of HBA2 expression with Neurons; G: Correlation of HBA2 expression with NK cell; H: Correlation of HBA2 expression with Regulatory T cells.

**Figure 11:**
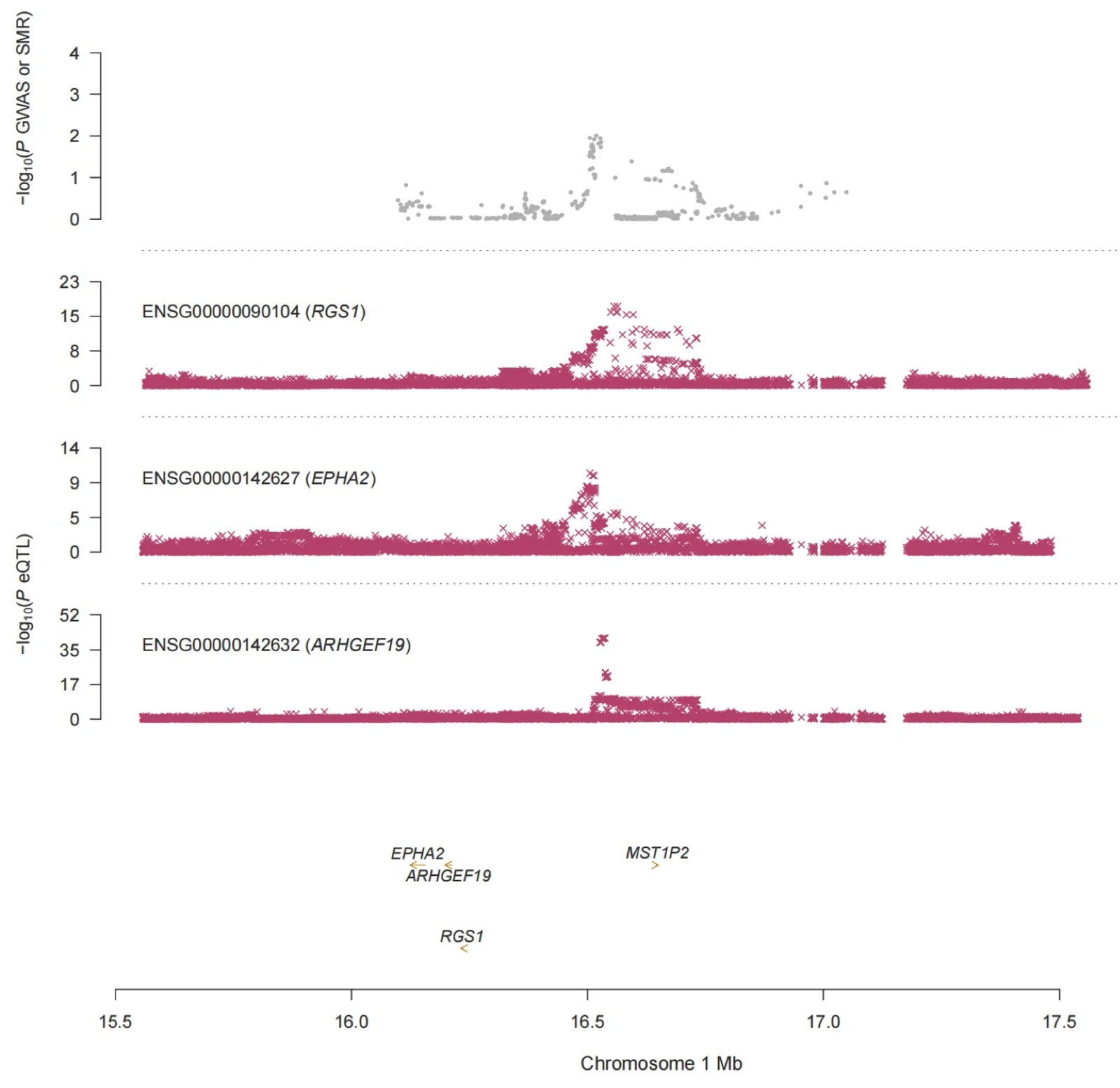
SMR analysis to validate hub genes.
