# Supplementary material for: The scPagwas analysis of single-cell sequencing combined with Bayesian deconvolution with SMR Reveals Tumor Heterogeneity and Predicts Immunotherapy Response in Neuroblastoma
Source: J Cancer. 2026 Sep 28;17(11):1853–71. doi: 10.7150/jca.138740 (PMC13686776; doi:10.7150/jca.138740)
Supplement: Supplementary file 1 [file jcav17p1853s1.pdf]

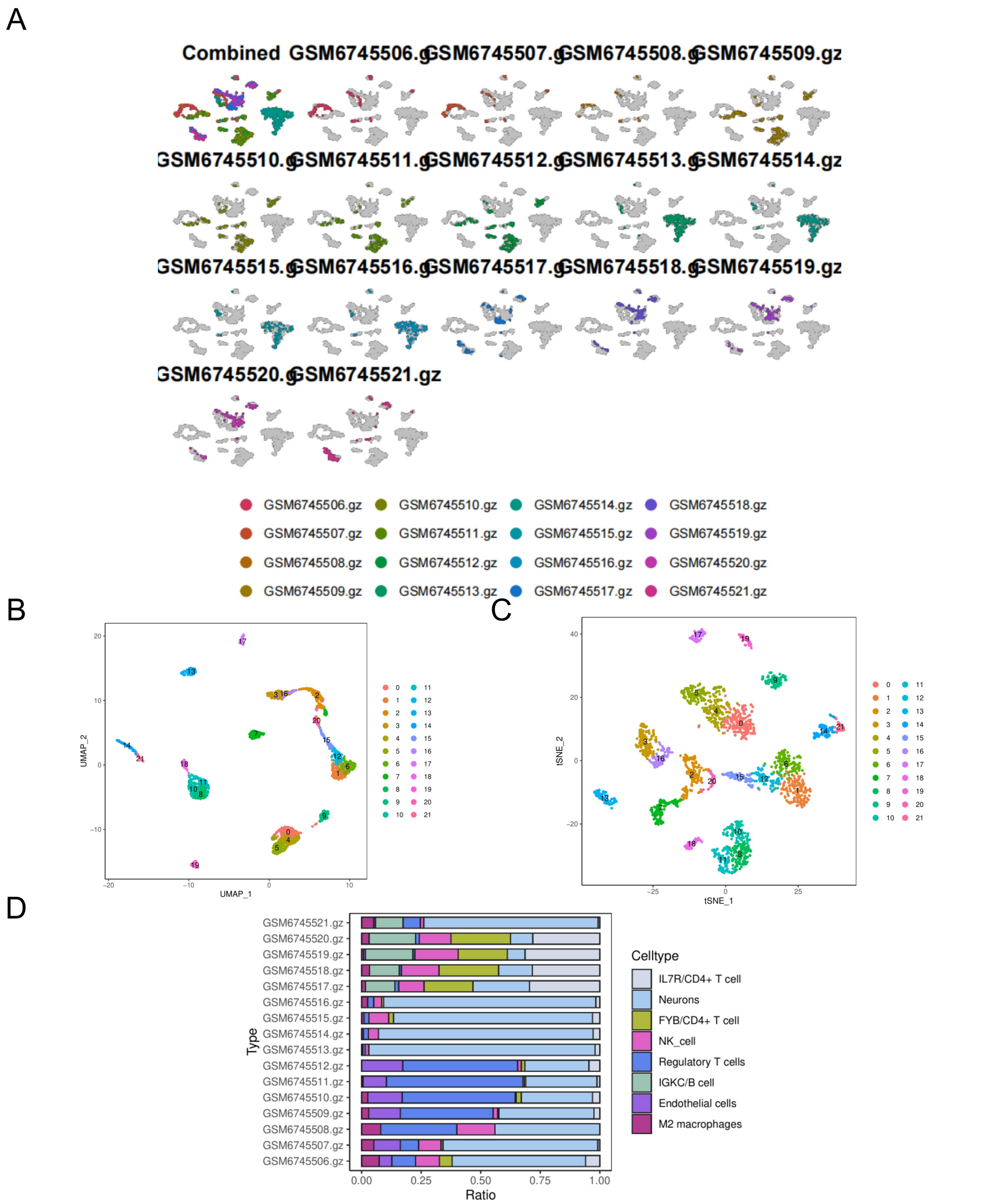

S1 Filtering and Annotation of scRNA-Seq Data. **A:**PCA showed a clear separation of cells in NB; **B-C:**The core cells were classified into 21 independent cell clusters using the Uniform Manifold Approximation and Projection (UMAP) (B) and t-distributed Stochastic Neighbor Embedding (t-SNE) algorithm (C); **D:**The percentage of each cell in each sample

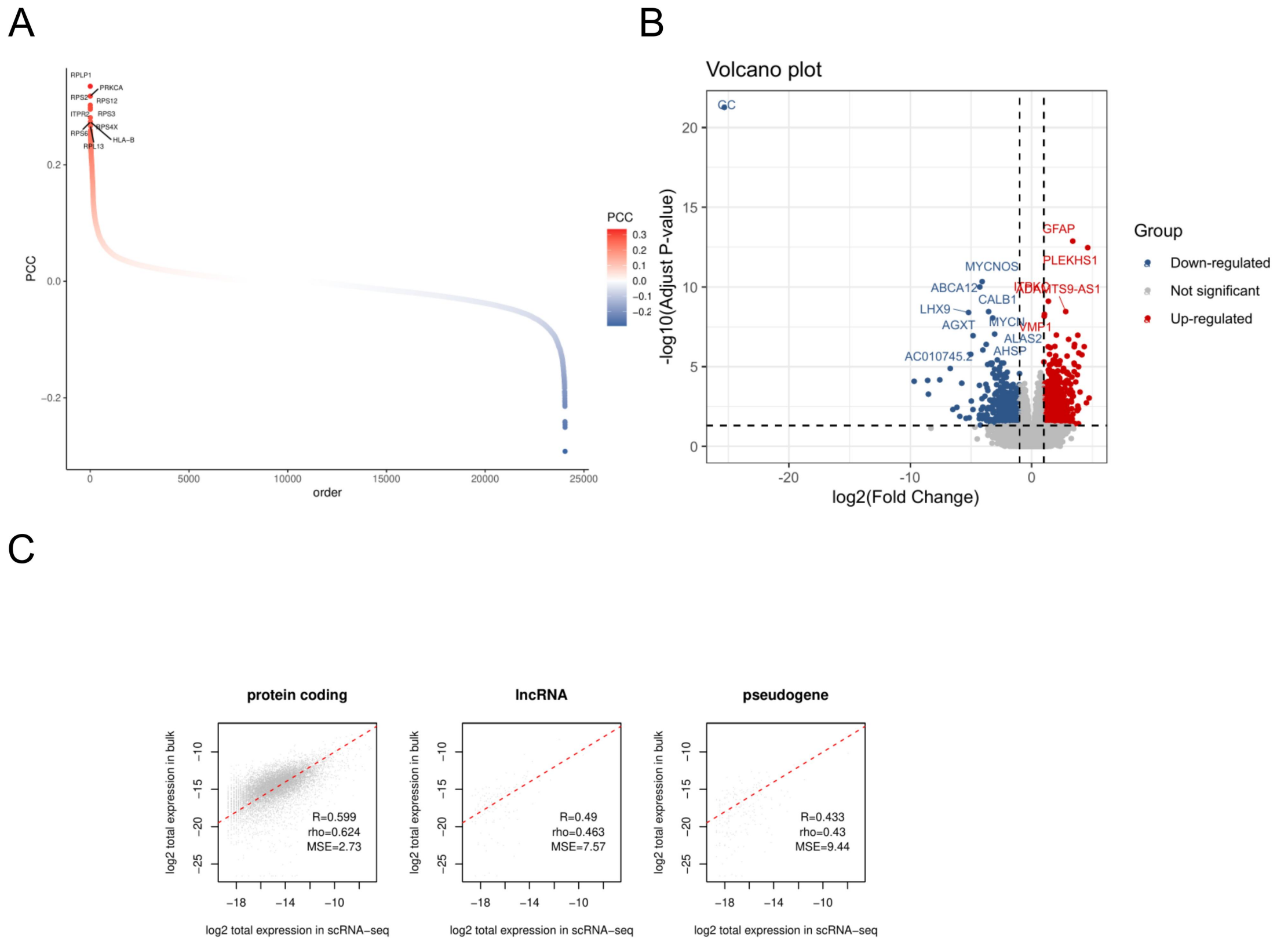

S2.scPagwas analysis and Bayesian inverse convolution analysis.**A:**Trait-related genes in neuroblastoma;**B:**Differentially expressed genes from RNA-Seq data;**C:**Bayesian inverse convolution analysis

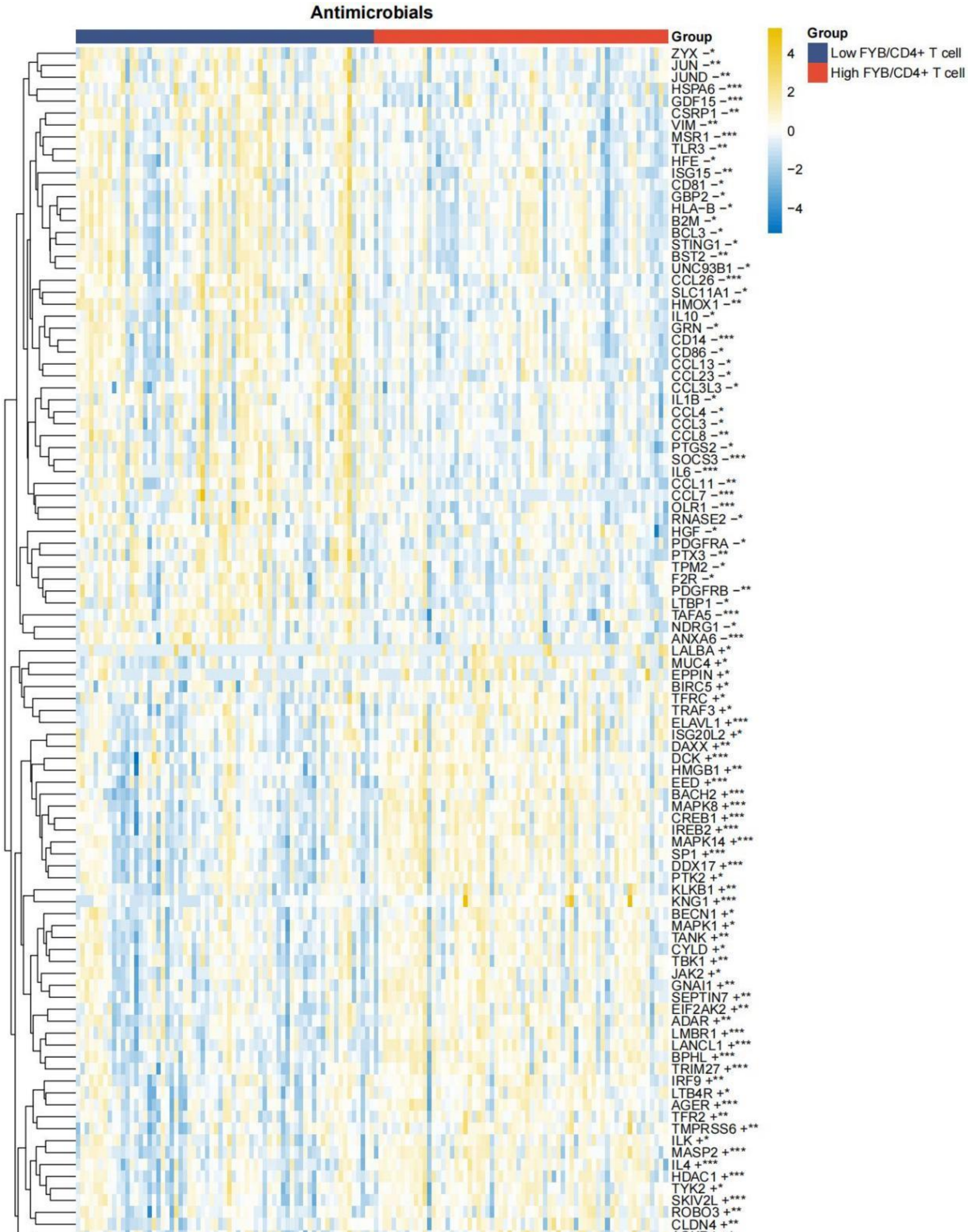

S3. FYB/CD4+ T cell with Antimicrobials gene set

*P＜0.05；**P＜0.01;***P＜0.001

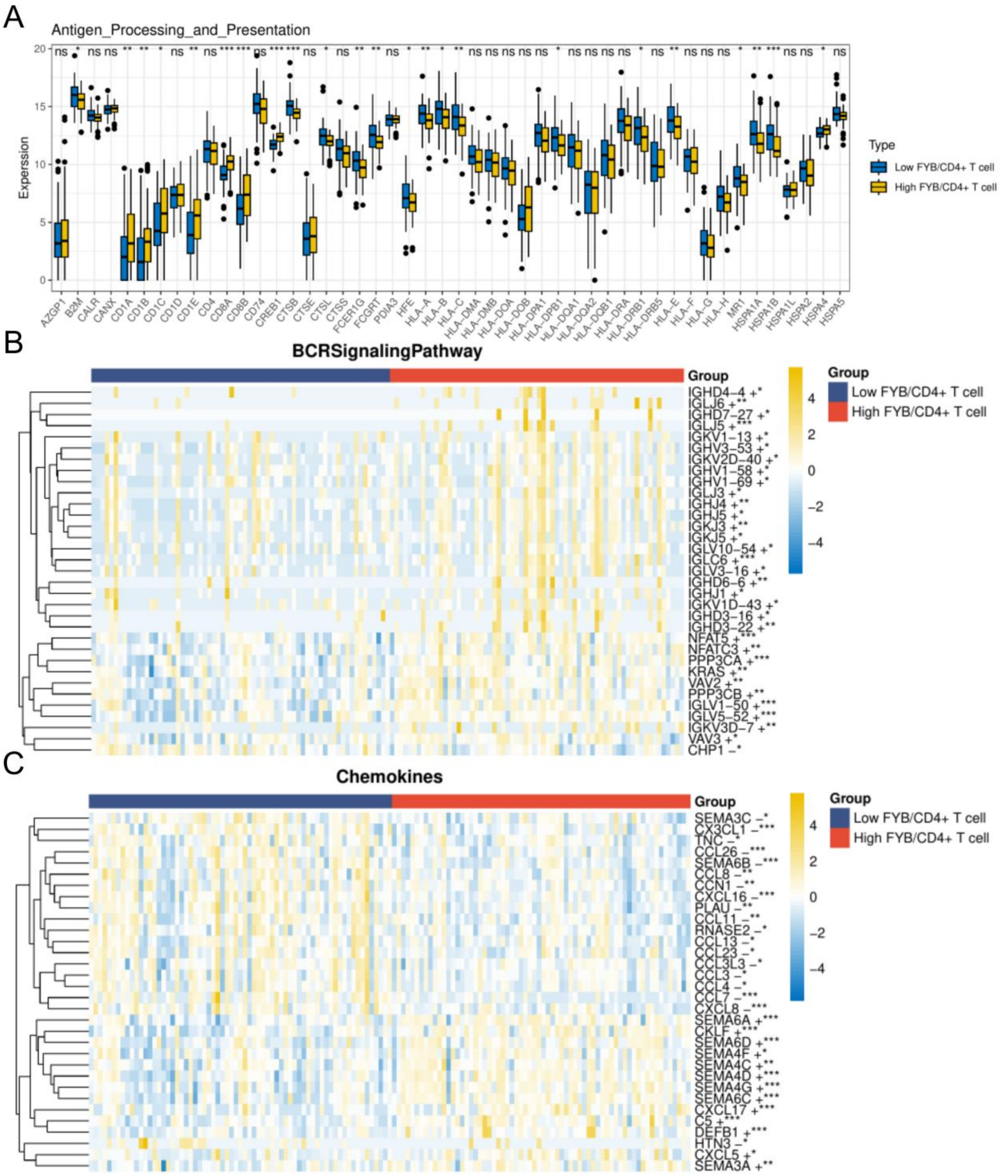

S4.FYB/CD4+ T cell and other immune gene sets(1) **A:**FYB/CD4+ T cell with Antigen Processing and Presentation gene set;**B:**FYB/CD4+ T cell with BCRSignalingPathway gene set;**C:**FYB/CD4+ T cell with Chemokines gene set

*P＜0.05；**P＜0.01;***P＜0.001

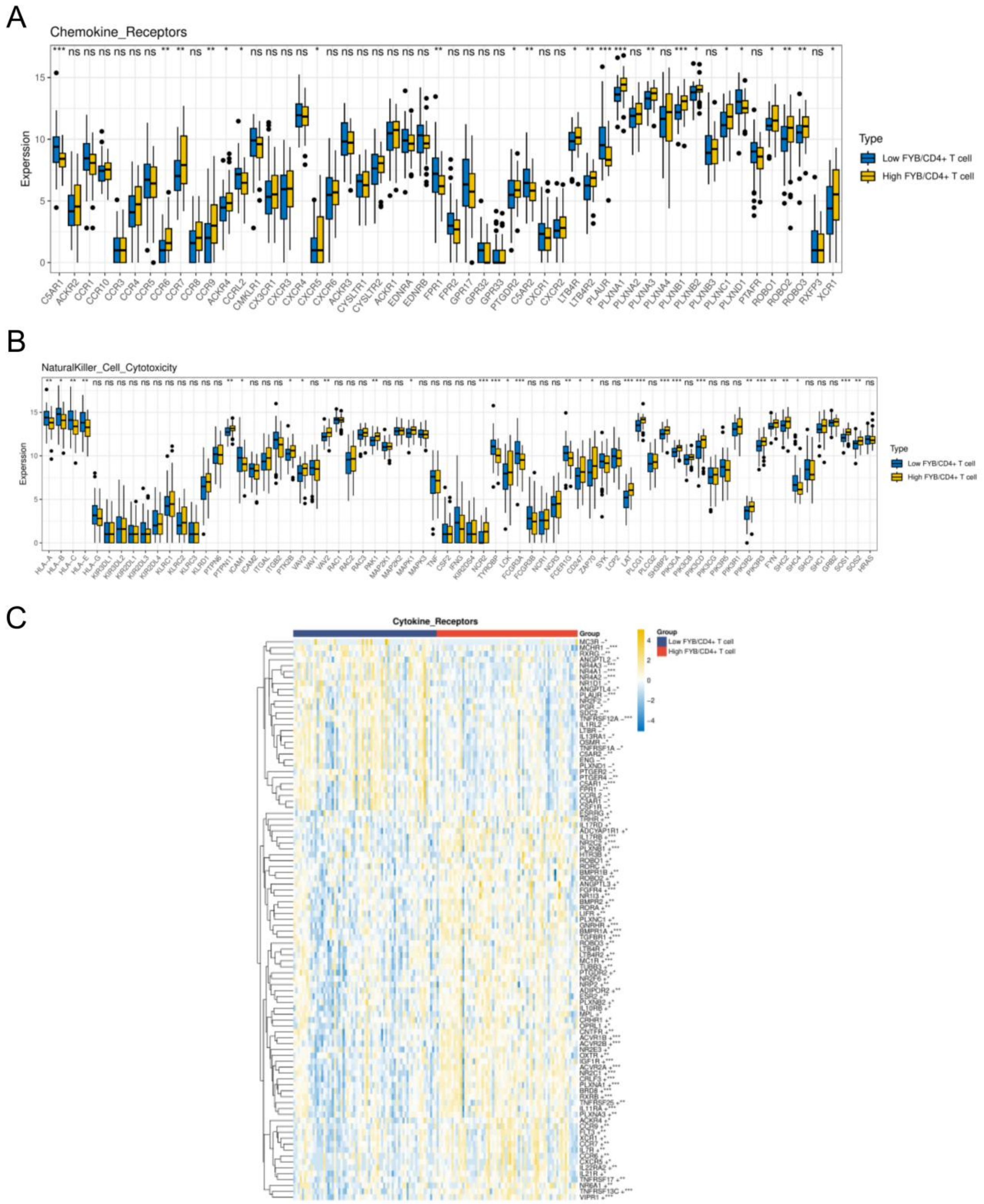

S5. FYB/CD4+ T cell with Chemokines gene set(2) **A:**FYB/CD4+ T cell with Chemokine Receptors gene set;**B:**FYB/CD4+ T cell and NaturalKiller Cell Cytotoxicity gene set;**C:**FYB/CD4+ T cell with Cytokine Receptors gene set.

*P＜0.05；**P＜0.01;***P＜0.001

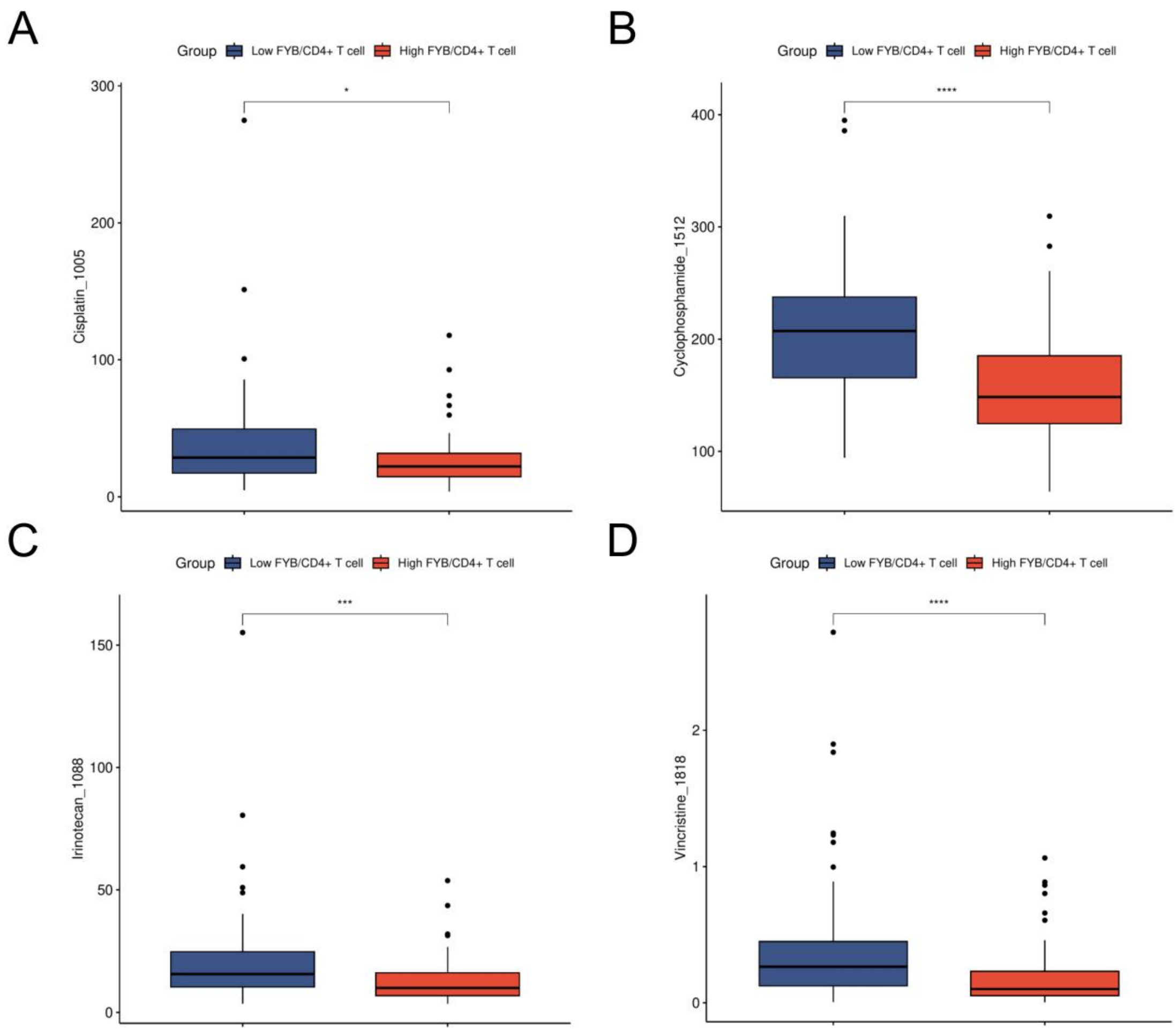

S6. Drug sensitivity analysis. **A:**Drug sensitivity analysis of Cisplatin;**B:**Drug susceptibility analysis of Cyclophosphamide;**C:**Drug susceptibility analysis of Irinotecan;**D:**Drug susceptibility analysis of Vincristine.

*P＜0.05；**P＜0.01;***P＜0.001,****P＜0.0001

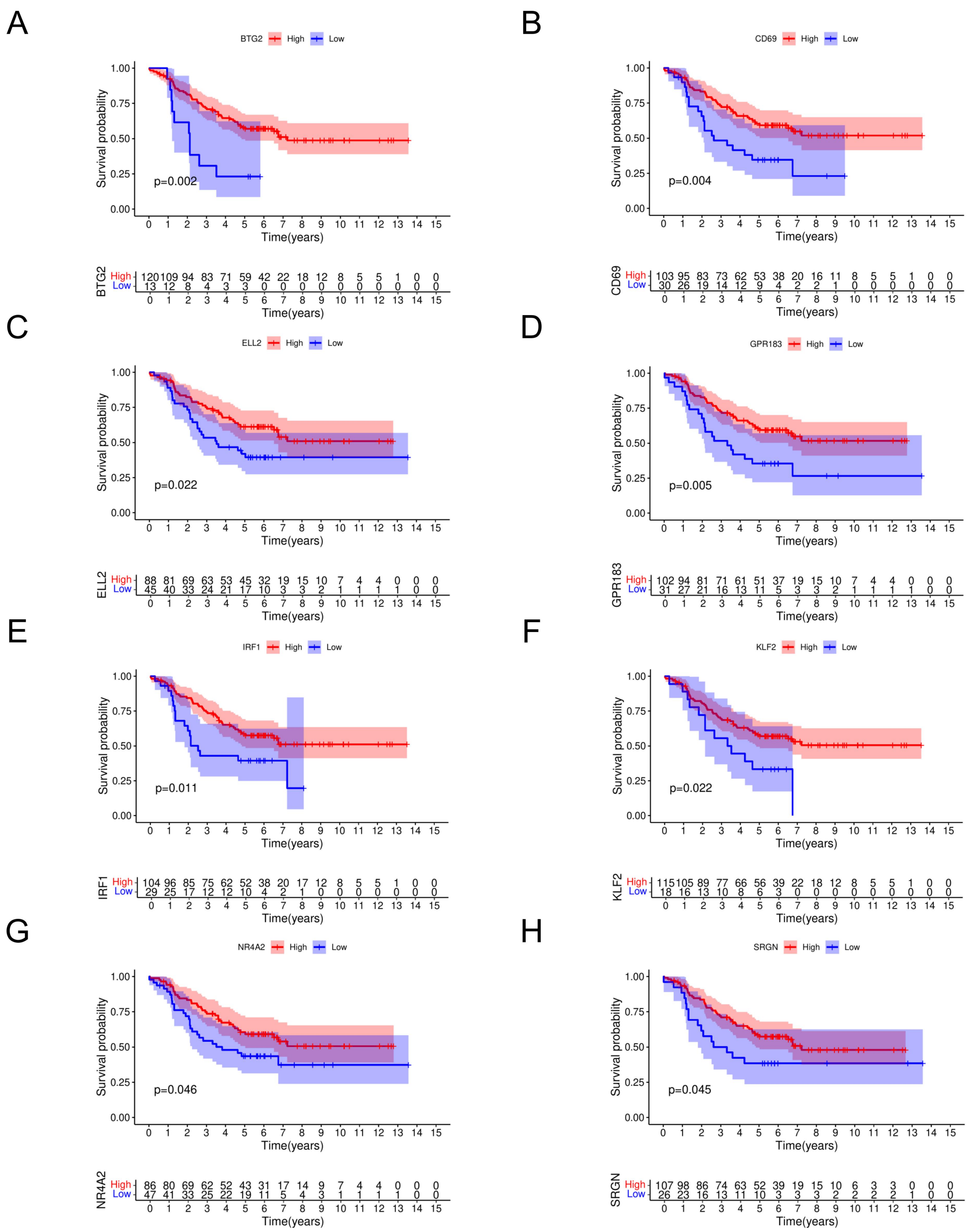

S7.K-M curve of the Hub gene. **A:**K-M curve of BTG2.;**B:**K-M curve of CD69;**C:**K-M curve of ELL2;**D:**K-M curve of GPR183;**E:**K-M curve of IRF1;**F:**K-M curve of KLF2;**G:**K-M curve of NR4A2;**H:**K-M curve of SRGN.

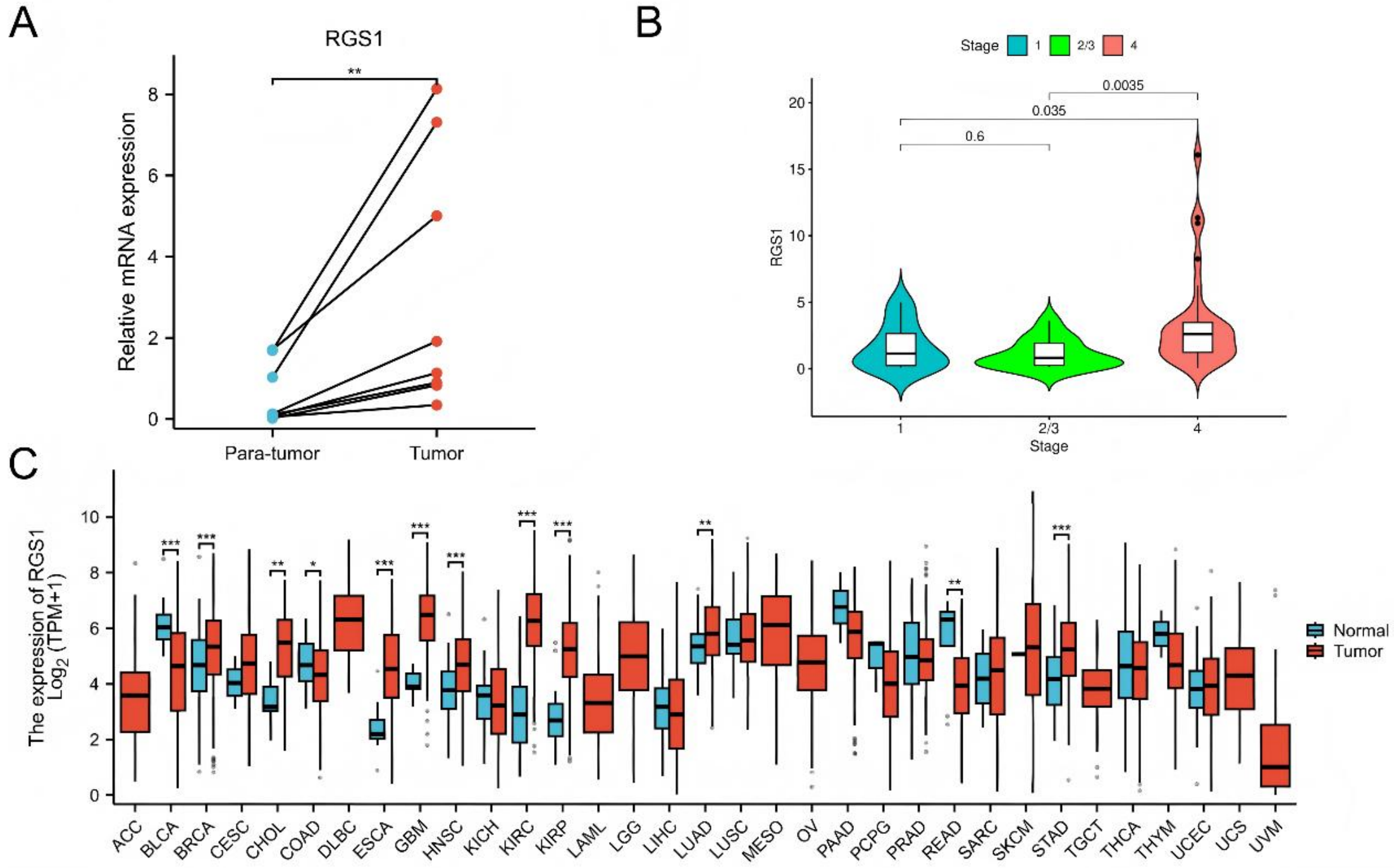

S8.RGS1 expression in tumors. A: The relative expression level of RGS1 in the paired samples of 8 cases showed differences; B: Gene expression and tumor staging; C: Pan-cancer analysis of RGS1.

*P＜0.05；**P＜0.01;***P＜0.001,****P＜0.0001

Supplementary Table1

	Primer Sequences(5'to3')
GAPDH-F	GTCTCCTCTGACTTCAACAGCG
GAPDH-R	ACCACCCTGTTGCTGTAGCCAA
RGS1-F	TCTTCTCTGCTAACCCAAAGGA
RGS1-R	TGCTTTACAGGGCAAAAGATCAG
